# Measurable therapeutic antibody in serum as potential predictive factor of response to anti-CD38 therapy in non-IgG-k myeloma patients

**DOI:** 10.1186/s40164-024-00547-x

**Published:** 2024-08-06

**Authors:** Emilia Gigliotta, Federica Plano, Giusy Corsale, Anna Maria Corsale, Cristina Aquilina, Maria Speciale, Andrea Rizzuto, Enrica Antonia Martino, Dario Leotta, Antonio Giovanni Solimando, Roberto Ria, Massimo Gentile, Sergio Siragusa, Cirino Botta

**Affiliations:** 1 Department of Health Promotion, Mother and Child Care, Internal Medicine and Medical Specialties, University of Palermo, Palermo, Italy; 2 Hematology Unit, Azienda Ospedaliera Annunziata, Cosenza, Italy; 3 Unit of Internal Medicine “Guido Baccelli”, Department of Precision and Regenerative Medicine and Ionian Area (DiMePRe-J), University of Bari “Aldo Moro” Medical School, Bari, Italy; 4 Department of Pharmacy, Health and Nutritional Science, University of Calabria, Rende, Italy

**Keywords:** Multiple myeloma, Anti-CD38 therapies, Body mass index, Inflammation

## Abstract

Multiple myeloma (MM) is a hematologic malignancy characterized by abnormal plasma cell proliferation in the bone marrow. Recent advancements in anti-CD38 monoclonal antibody therapies, such as daratumumab and isatuximab, have significantly improved MM patient survival. However, the lack of predictive factors of response to these therapies remains a challenge. Notably, anti-CD38 antibodies can interfere with laboratory tests, complicating response assessment. We conducted a retrospective study to evaluate the association between the appearance of positive IgGk (therapeutic antibody) on immunofixation/immunosubtraction (IF) and clinical parameters in 87 non-IgGk MM patients treated with anti-CD38 therapy. Positive IgGk IF was observed in 42 patients after a median of three treatment courses. Patients with positive IgGk IF had higher rates of complete/very good partial responses (p = 0.03) and improved progression-free survival (median not reached vs. 21.83 months, p < 0.01). High BMI (p = 0.03), higher hemoglobin (p = 0.02), lower CRP (p = 0.04), and lower monoclonal protein levels (p = 0.03) were associated with positive IgGk IF. Our findings suggest that monitoring therapeutic antibody appearance on IF may predict and optimize anti-CD38 therapy in MM. Potential explanations include the impact of patient factors (e.g. BMI) on drug pharmacokinetics, the relationship between antibody levels and immune response, and the influence of tumor biology. Further research is needed to elucidate the underlying mechanisms and clinical utility of this biomarker. Nonetheless, our results highlight the importance of considering therapeutic antibody detection when interpreting laboratory tests and managing MM patients receiving anti-CD38 therapies.

**Supplementary Information:**

The online version contains supplementary material available at 10.1186/s40164-024-00547-x.

## To the editor,

**Fig. 1:**
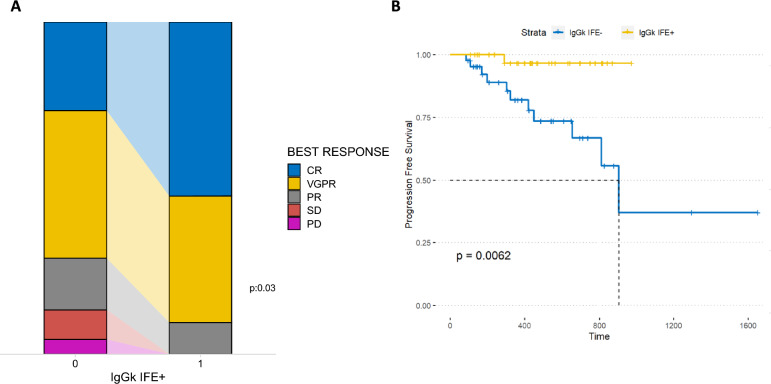
Response rate (**A**) and progression-free survival (PFS) (**B**) of the patients analyzed based on the appearance of IgGk positive immunofixation

**Fig. 2:**
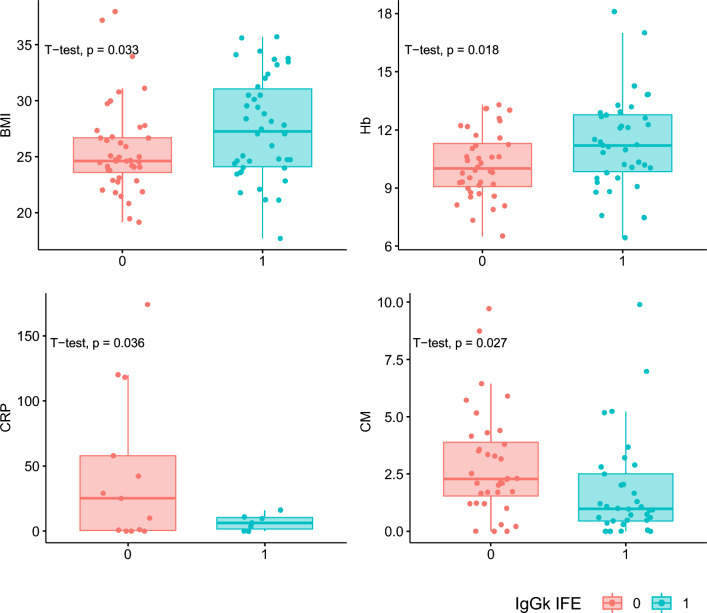
Dot plot of laboratory parameters significantly associated with the appearance of IgGk positive immunofixation (*BMI* body mass index, *Hb* hemoglobin, *CRP* C-reactive protein, *CM* monoclonal component)

Multiple myeloma (MM) is a hematologic malignancy characterized by the proliferation of abnormal plasma cells within the bone marrow [1] which has seen promising advancements with monoclonal antibodies targeting the CD38 protein (daratumumab/isatuximab) [2, 3]. While these therapies have demonstrated substantial efficacy in enhancing MM patient survival, the lack of predictive factors for response to anti-CD38 therapy poses a considerable challenge. Furthermore, it is noteworthy that these antibodies may migrate along with serum proteins once treatment commences, potentially complicating the interpretation of laboratory tests [4], being detectable within serum protein electrophoresis, and/or in serum immunofixation/immunosubtraction (IF) [4]. This phenomenon can pose challenges in accurately assessing the quality of the therapeutic response achieved. On the other hand, while often underestimated, the detection or absence of these antibodies in laboratory tests could serve as a marker for the concentration/presence of the antibody in the patient. To explore the latter point, we conducted a retrospective study (within the MMVision and VISIUMM studies, approved by our internal ethical committee with the number 02/2022 and 1300 12/2023) to evaluate the association between the appearance of positive IgGk (i.e. the therapeutic antibody) at IF (measured at day 1 of each cycle) and clinical parameters/outcome measures in 87 non-IgGk MM patients treated with daratumumab or isatuximab in three different hematology centers. The patient cohort included 34 IgA (22 kappa and 12 lambda), 38 IgG lambda, 1 IgD, 1 IgM, 10 light chains, and 3 low/non-secreting MM. Main patients’ characteristics and treatment schedules are reported in Table 1 and in Supplementary Table 1. Interestingly, a positive IgGk IF was observed in 42/87 patients, after a median of three treatment courses. Our results demonstrated a significant association between positive IgGk IF and a higher rate of CR/VGPR responses to anti-CD38 therapy (chi-square p = 0.03) (Fig. 1A). Furthermore, we investigated whether these results could impact patient outcomes. Interestingly, we found that patients who developed IgGk IF + had improved progression-free survival (PFS) compared to those who did not (median PFS not reached, versus 21.83 months respectively, HR: 0.10, p < 0.01) (Fig. 1B and Supplementary Fig. 1A, the latter showing results excluding patients who received fixed-duration Daratumumab). Additionally, this variable remained significant in a multivariate Cox regression model (Supplementary Fig. 1B). Of note, survival comparison between IgGk and non-IgGk MM patients showed no differences in terms of PFS (IgGk MM n = 54, supplementary Fig. 2A). Next, we explored clinical and laboratory parameters for their association with positive IF appearance (all variables and analysis, including administration routes comparisons, reported in Supplementary Table 2 and supplementary Fig. 3 and 4), with a high BMI (p = 0.03), higher hemoglobin levels (p = 0.02), lower CRP levels (p = 0.04), and lower monoclonal component levels (p = 0.03) emerging as the most significantly associated factors (Fig. 2). In multivariate analysis (generalized linear model, supplementary Fig. 2B), only BMI evaluation maintained its statistical significance (interestingly, hemoglobin and monoclonal protein resulted highly correlated, supplementary Fig. 5), thus supporting the idea that these results could depend, at least in part, on drug bio-availability (of note, only 10 patients received the majority of treatment courses in an iv route). Along the same line, iv Daratumumab already demonstrated an increased half-life in patients with a BMI > 30 [5]. Systemic absorption of daratumumab, as well as other monoclonal antibodies (mAbs) after subcutaneous injection, occurs primarily through the lymphatic system, influenced by factors such as temperature, pH, interstitial fluid composition, lymphatic capillary density, and molecular characteristics including size, charge density, and immunogenicity [6, 7]. FcRn and non-specific binding affect transport, while presystemic catabolism may limit mAb availability in the central compartment [8]. Recent studies suggest that obese patients have lower blood volume per kilogram and lower IgG clearance, which could extend the half-life of mAbs [9, 10]. These mechanisms, alongside the increased feasibility of subcutaneous administration in obese patients, may improve drug bioavailability and enhance daratumumab detection by immunofixation [5, 8–10]. Overall, the appearance of therapeutic antibodies in serum, as detected by immunofixation, likely reflects a combination of factors, including drug pharmacokinetics, immune response dynamics, and tumor biology [1]. Our findings suggest that checking these parameters may be crucial in predicting and monitoring responses to anti-CD38 therapy as well as optimizing treatment strategies for MM patients. Further research is needed to elucidate the specific mechanisms underlying this phenomenon and its potential role as a predictive factor for treatment response in multiple myeloma patients.

**Table 1:** Main patients characteristics at baseline

Heavy chain	IGG: 38 IGA: 34 IGD: 1 IGM: 1 LC: 10 OTHERS: 3
Light chain	K: 26 L: 58 N/A: 3
ISS	ISS 1: 28 ISS 2: 26 ISS 3: 31 N/A: 2
Associated treatment (Anti-CD38 +)	KD: 2 RD: 2 VD: 3 VMP: 5 VTD: 12
Treatment line	I: 53 II: 24 > II: 10
BMI (Mean)	26.56

### Supplementary Information

Additional file 1: Figure 1. (A) Survival comparison in terms of PFS between non-IgGk multiple myeloma (MM) patients who become positive for IgGk by IF+. We excluded 20 MM patients treated with the Dara-VTD regimen to avoid potential interference related to the fixed-duration treatment. (B) Forest plot reporting the results of the Cox regression analysis conducted on the seven variables that passed the univariate test (p-value < 0.1). Among these, the emergence of IgGk IF+ was identified as the only independent variable significantly associated with survival. Figure 2. Survival comparison between IgGk and non-IgGk MM patients (A) used to demonstrates that the isotype does not affect PFS; forest plot of hazard ratios of laboratory parameters associated with positive IgGk immunofixation appearance, multivariate analysis (B). Figure 3. Sankey plot showing response rates based on the appearance of IgGk+ at the IF across four distinct subgroups: patients who received only subcutaneous (sc) administration of anti-CD38 antibodies, those who initially received intravenous (iv) administration before switching to the sc route, patients who achieved the best response (BR) during sc treatment, and those who achieved BR during iv administration. Figure 4. Dot plot of laboratory parameters significantly associated with the appearance of IgGk positive immunofixation in the overall populations, now evaluated in the four different scenario as previously described (BMI: body mass index, Hb: hemoglobin, CRP: C-reactive protein, CM: monoclonal component). Figure 5. Correlation plot of laboratory parameters in the analyzed population.

Additional file 2: Table 1. List of the main patients’ characteristics grouped according to the appearance of IgGk IF+

Additional file 3: Table 2. Complete list of analyzed laboratory parameters, mean values for IgGk+ and IgGk- populations, and their correlation with the presence of a positive IgGk immunofixation.

## Data Availability

No datasets were generated or analysed during the current study.

## Abbreviations

ADCC: Antibody-dependent cellular cytotoxicity
ADCP: Antibody-dependent cellular phagocytosis
BMI: Body mass index
CDC: Complement-dependent cytotoxicity
CR: Complete response
CRP: C reactive protein
IF: Immunofixation/immunosubtraction
MM: Multiple myeloma
PFS: Progression-free survival
VGPR: Very good partial response

## References

[1] Plano F, et al. Monoclonal Gammopathies and the bone marrow microenvironment: from bench to bedside and then back again. Hematol Rep. 2023;15(1):23–49. 36648882 10.3390/hematolrep15010004 PMC9844382

[2] Dimopoulos MA, et al. Corrigendum to “multiple myeloma: EHA-ESMO clinical practice guidelines for diagnosis, treatment and follow-up.” Ann Oncol. 2022;33(1):117. 34857439 10.1016/j.annonc.2021.10.001

[3] Botta C, et al. Network meta-analysis of randomized trials in multiple myeloma: efficacy and safety in frontline therapy for patients not eligible for transplant. Hematol Oncol. 2022;40(5):987–98. 35794705 10.1002/hon.3041 PMC10084226

[4] Jimenez A, et al. Characteristics of isatuximab-derived interference in serum protein electrophoresis and immunofixation, and an absence of sustained in vivo interference due to belantamab mafodotin and denosumab. Clin Biochem. 2024. 10.1016/j.clinbiochem.2024.110761. 38565341 10.1016/j.clinbiochem.2024.110761

[5] Abdallah N, et al. Tracking daratumumab clearance using mass spectrometry: implications on M protein monitoring and reusing daratumumab. Leukemia. 2022;36(5):1426–8. 35091659 10.1038/s41375-021-01501-0 PMC9061287

[6] Usmani SZ, et al. Subcutaneous delivery of daratumumab in relapsed or refractory multiple myeloma. Blood. 2019;134(8):668–77. 31270103 10.1182/blood.2019000667 PMC6754719

[7] Rahimi E, et al. Transport and lymphatic uptake of monoclonal antibodies after subcutaneous injection. Microvasc Res. 2022;139: 104228. 34547346 10.1016/j.mvr.2021.104228

[8] Sanchez-Felix M, et al. Predicting bioavailability of monoclonal antibodies after subcutaneous administration: open innovation challenge. Adv Drug Deliv Rev. 2020;167:66–77. 32473188 10.1016/j.addr.2020.05.009

[9] Erstad BL, Barletta JF. Implications of obesity for drug administration and absorption from subcutaneous and intramuscular injections: a primer. Am J Health Syst Pharm. 2022;79(15):1236–44. 35176754 10.1093/ajhp/zxac058

[10] Li Z, et al. Effects of body mass and age on the pharmacokinetics of subcutaneous or hyaluronidase-facilitated subcutaneous immunoglobulin G in primary immunodeficiency diseases. J Clin Immunol. 2023;43(8):2127–35. 37773562 10.1007/s10875-023-01572-x PMC10661727

