# Supplementary material for: Measurable therapeutic antibody in serum as potential predictive factor of response to anti-CD38 therapy in non-IgG-k myeloma patients
Source: Exp Hematol Oncol. 2024 Aug 6;13:82. doi: 10.1186/s40164-024-00547-x (PMC11302264; doi:10.1186/s40164-024-00547-x)

Supplementary Figure 1

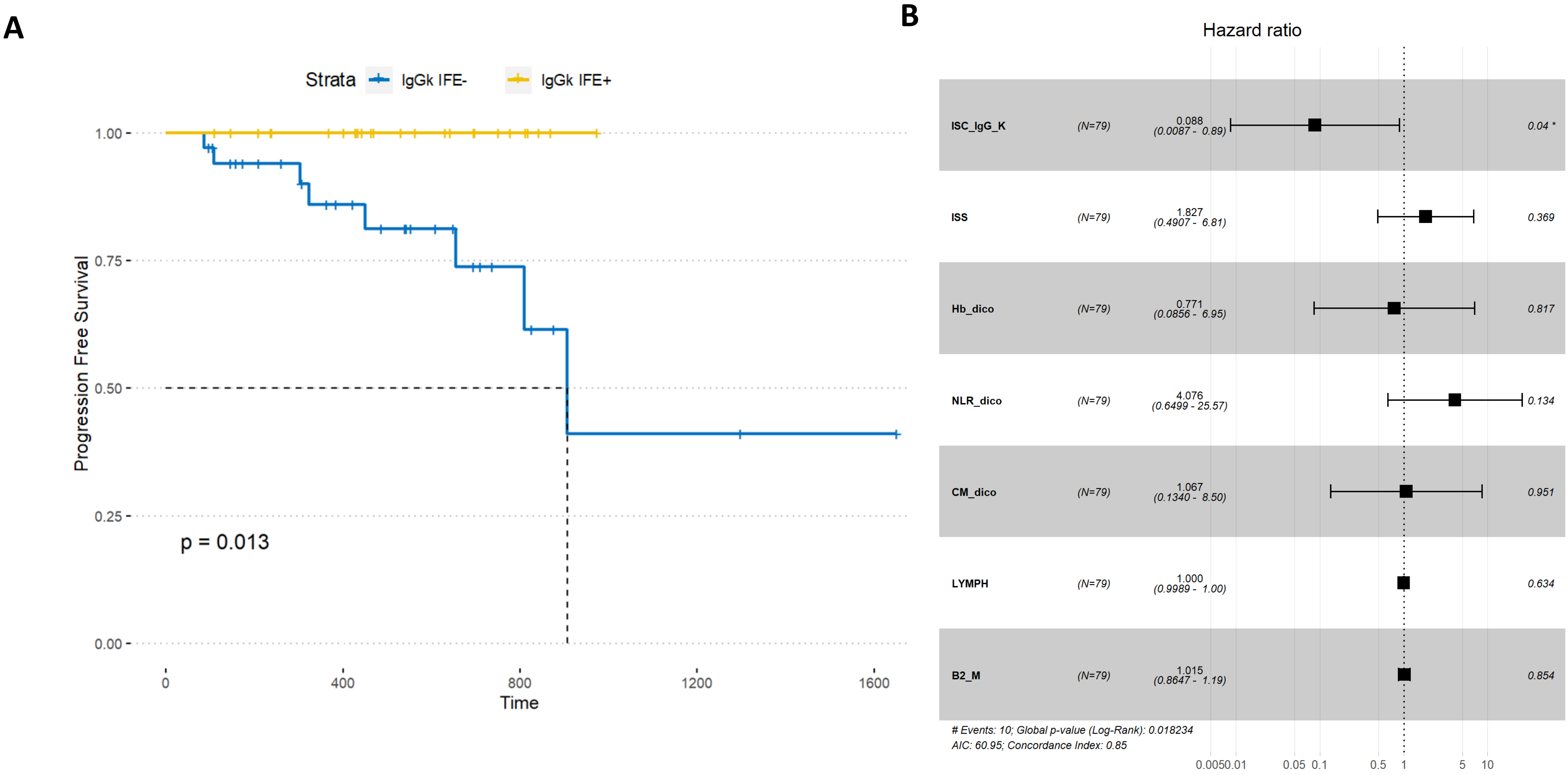

Supplementary Figure 2

A

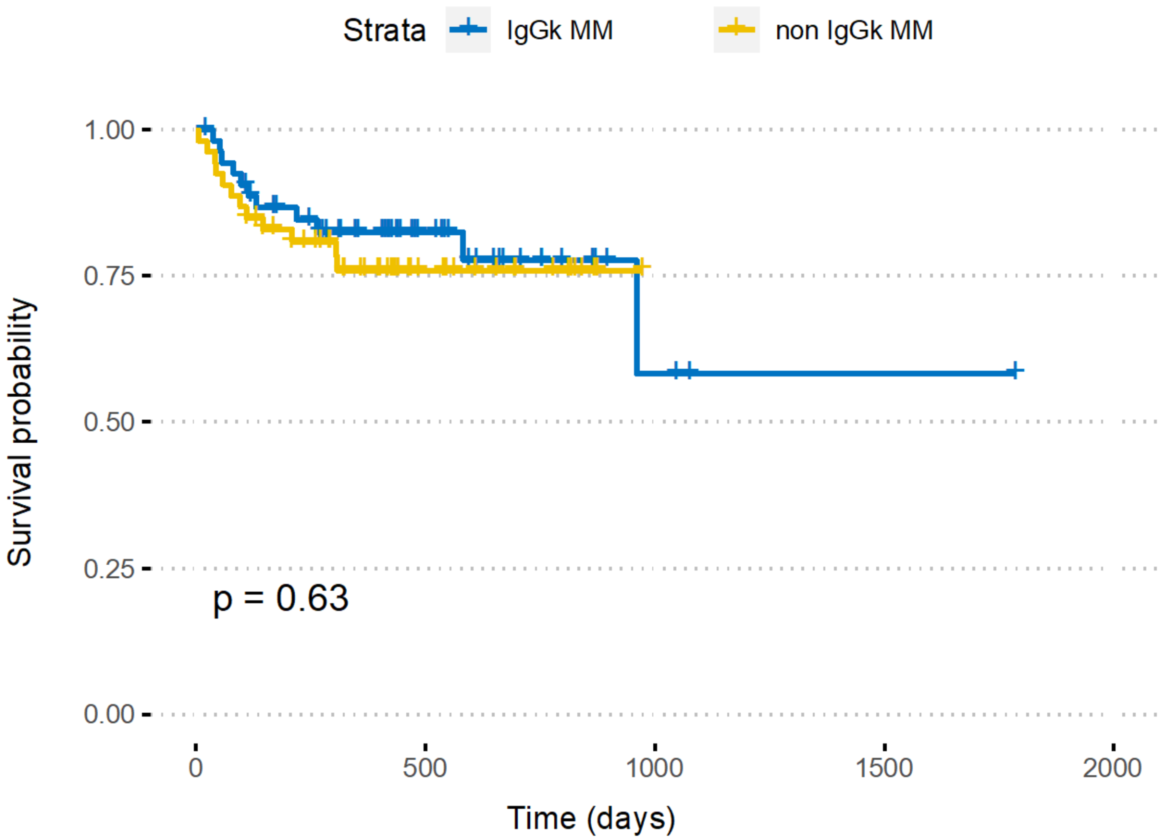

B

Variable	N	Estimate	p
WEIGHT	65	0.01 (−0.02, 0.03)	0.66
HEIGHT	65	0.01 (−0.02, 0.04)	0.48
SURFACE	65	−1.04 (−4.35, 2.26)	0.54
BMI	65	0.04 (0.00, 0.08)	0.05
HB	65	0.03 (−0.04, 0.10)	0.44
NEUT	65	−0.00 (−0.00, 0.00)	0.36
LYMPH	65	0.00 (−0.00, 0.00)	0.69
MONO	65	0.00 (−0.00, 0.00)	0.39
CM	65	−0.05 (−0.11, 0.02)	0.15

Supplementary Figure 3

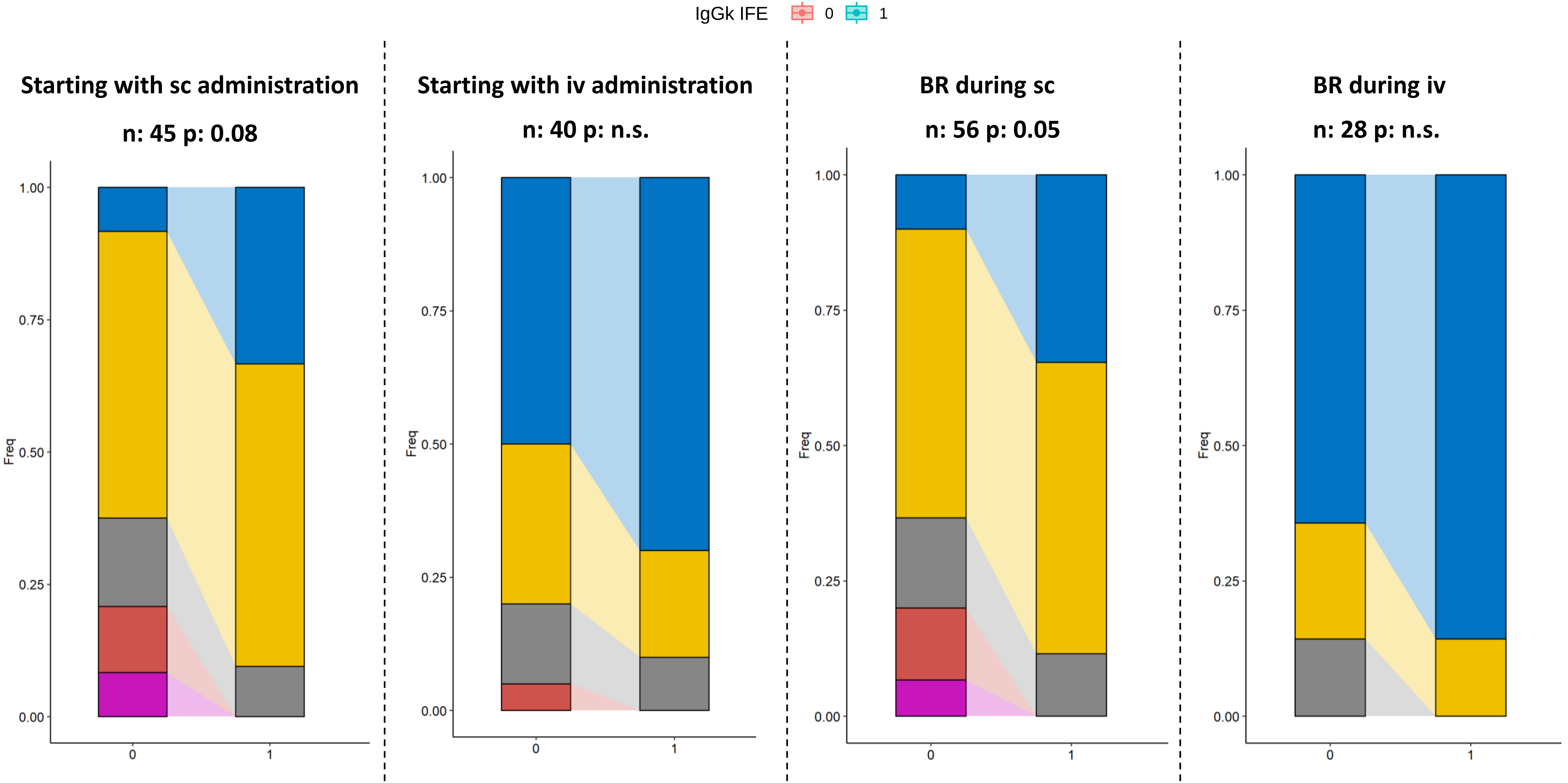

Supplementary Figure 4

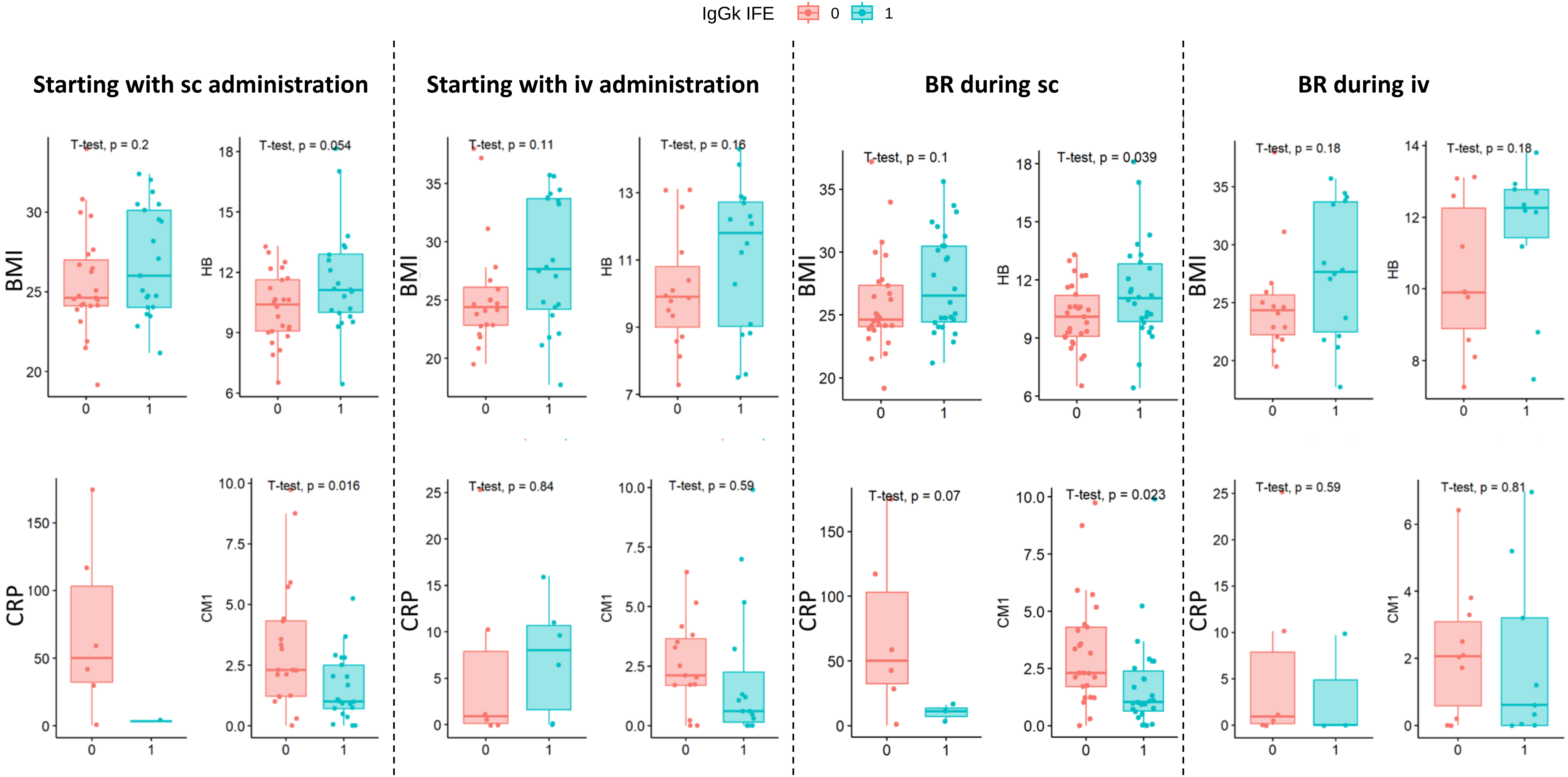

Supplementary Figure 5

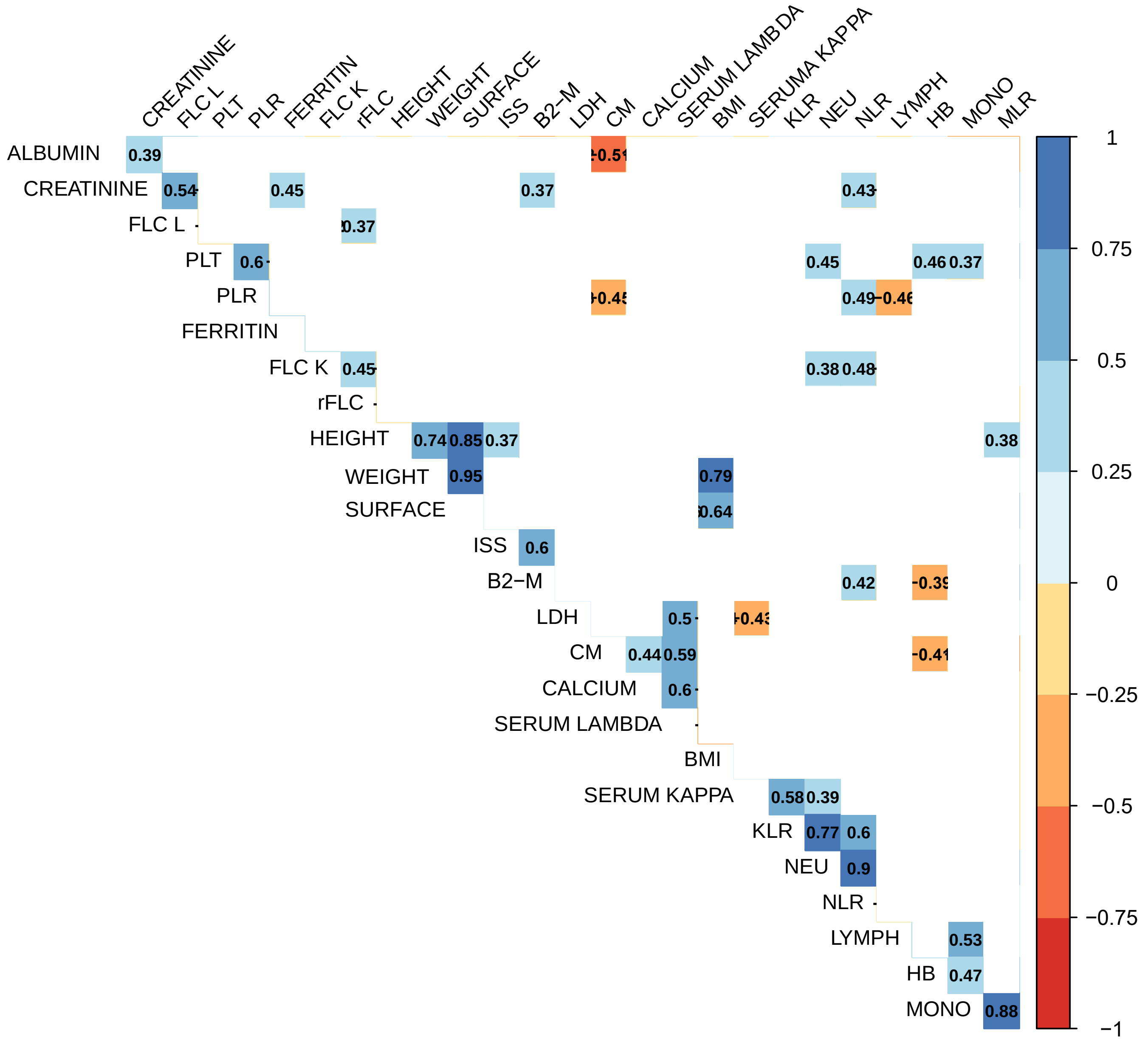

Supplement: Supplementary file 1 — Additional file 1: Figure 1. (A) Survival comparison in terms of PFS between non-IgGk multiple myeloma (MM) patients who become positive for IgGk by IF+. We excluded 20 MM patients treated with the Dara-VTD regimen to avoid potential interference related to the fixed-duration treatment. (B) Forest plot reporting the results of the Cox regression analysis conducted on the seven variables that passed the univariate test (p-value < 0.1). Among these, the emergence of IgGk IF+ was identified as the only independent variable significantly associated with survival. Figure 2. Survival comparison between IgGk and non-IgGk MM patients (A) used to demonstrates that the isotype does not affect PFS; forest plot of hazard ratios of laboratory parameters associated with positive IgGk immunofixation appearance, multivariate analysis (B). Figure 3. Sankey plot showing response rates based on the appearance of IgGk+ at the IF across four distinct subgroups: patients who received only subcutaneous (sc) administration of anti-CD38 antibodies, those who initially received intravenous (iv) administration before switching to the sc route, patients who achieved the best response (BR) during sc treatment, and those who achieved BR during iv administration. Figure 4. Dot plot of laboratory parameters significantly associated with the appearance of IgGk positive immunofixation in the overall populations, now evaluated in the four different scenario as previously described (BMI: body mass index, Hb: hemoglobin, CRP: C-reactive protein, CM: monoclonal component). Figure 5. Correlation plot of laboratory parameters in the analyzed population. [file 40164_2024_547_MOESM1_ESM.pdf]
