# Supplementary material for: Measurable therapeutic antibody in serum as potential predictive factor of response to anti-CD38 therapy in non-IgG-k myeloma patients
Source: Exp Hematol Oncol. 2024 Aug 6;13:82. doi: 10.1186/s40164-024-00547-x (PMC11302264; doi:10.1186/s40164-024-00547-x)
Supplement: Supplementary file 2 — Additional file 2: Table 1. List of the main patients’ characteristics grouped according to the appearance of IgGk IF+ [file 40164_2024_547_MOESM2_ESM.docx]

| Main patients characteristics at baseline according to IFE | |  |
| --- | --- | --- |
|  | IgGk + (42) | IgGk – (45) |
| Heavy chain | IgG: 16  IgA: 18  IgD: 1  IgM: 0  LC: 5  Others: 2 | IgG: 22  IgA: 16  IgD: 0  IgM: 1  LC: 5  Others: 1 |
| Light chain | K: 13  L: 28  N/A: 1 | K: 13  L: 30  N/A: 2 |
| ISS | ISS 1: 15  ISS 2: 13  ISS 3: 13  N/A: 1 | ISS 1: 13  ISS 2: 13  ISS 3: 18  N/A: 1 |
| Associated treatment (anti-CD38+) | KD: 2  RD: 24  VD: 4  VMP:2  VTD: 10 | KD: 1  RD: 27  VD: 3  VMP: 4  VTD: 10 |
| Treatment line | I: 27  II: 11  >II: 4 | I: 26  II: 13  >II: 6 |

Supplementary Table 1
