# Supplementary material for: Measurable therapeutic antibody in serum as potential predictive factor of response to anti-CD38 therapy in non-IgG-k myeloma patients
Source: Exp Hematol Oncol. 2024 Aug 6;13:82. doi: 10.1186/s40164-024-00547-x (PMC11302264; doi:10.1186/s40164-024-00547-x)
Supplement: Supplementary file 3 — Additional file 3: Table 2. Complete list of analyzed laboratory parameters, mean values for IgGk+ and IgGk- populations, and their correlation with the presence of a positive IgGk immunofixation. [file 40164_2024_547_MOESM3_ESM.docx]

|  | IFE IgGk+ | IFE IgGk- | p value |
| --- | --- | --- | --- |
| ISS | 1.95 | 2.11 | 0.37 |
| BODY weight | 73.79 | 69.31 | 0.19 |
| height | 162.7 | 160.60 | 0.51 |
| body surface | 1.795 | 1.71 | 0.14 |
| BMI | 27.59 | 25.58 | **0.03** |
| HB | 11.35 | 10.21 | **0.02** |
| neut count | 3688 | 3665 | 0.97 |
| LYMph count | 1840 | 1590 | 0.21 |
| MONO COUNT | 846 | 472 | 0.07 |
| PLATeLETS | 213947 | 190854 | 0.31 |
| creatinine | 1.52 | 1.34 | 0.53 |
| albumin | 3.64 | 4.33 | 0.50 |
| b2-m | 5.85 | 4.72 | 0.43 |
| calcium | 9.38 | 9.49 | 0.66 |
| ldh | 173.5 | 189 | 0.50 |
| esr | 44.1 | 47.18 | 0.82 |
| crp | 6.63 | 44.49 | **0.04** |
| ferritin | 391.04 | 326 | 0.63 |
| serum k | 3.21 | 2.32 | 0.34 |
| serum l | 3.05 | 4.89 | 0.18 |
| flc k | 80.53 | 135.66 | 0.45 |
| flc l | 728.30 | 930.07 | 0.70 |
| cm | 1.73 | 2.91 | **0.03** |
| nlr | 2.33 | 2,82 | 0.31 |
| mlr | 0.43 | 3.51 | 0.37 |

Supplementary table 2
